# Supplementary material for: Learning curve and surgical outcome of robotic assisted colorectal surgery with ERAS program
Source: Sci Rep. 2022 Nov 29;12:20566. doi: 10.1038/s41598-022-24665-w (PMC9709162; doi:10.1038/s41598-022-24665-w)
Supplement: Supplementary file 2 — Supplementary Information 2. [file 41598_2022_24665_MOESM2_ESM.zip › RAL Raw data-Ñ[▒K/072/Patho_ñw╝╨░O▒Kñσ.pdf]

臺中榮民總醫院 臺中市臺灣大道四段1650號 病理部活體組織切片 臨床醫學實驗及病理診斷中心 檢驗(查)報告

[redacted]

Pathologic diagnosis:
1. Rectum, robotic low anterior resection --- Tailgut cyst, focal ruptured with xanthomatous inflammation and old hemorrhage.
2. Rectum, distal cut end --- Unremarkable.

Gross description:
The specimen consists of 1) a segment of rectum, 7 cm in length and 2.8 cm in diameter of proximal cut end, 2.3 cm in diameter of distal cut end, with attached perirectal soft tissue. There is a cystic lesion, 5x3.5x3.3 cm, noted at the wall of rectum and perirectal soft tissue. On cut, mucosa of rectum is not connected to the cystic lesion. There is blood clot noted inside the cyst. Other parts of specimen are unremarkable. 2) a piece of tan soft tissue, 2.8x2.1x1 cm, labeled as "distal cut end". Representative parts for section: A-B) bilateral cut ends C-E) lesion F) non-lesion site G) specimen 2. Blue ink indicated serosa; yellow ink indicated radial margin; green ink indicated mucosa near the lesion site.

Note:
The diagnosis is concurred in intradepartment consensus meeting on 2021/04/14.
#T-59600_2 #M-26720_2 0 1100 00CM00

已電子簽章

警告：本單僅供醫師參考使用，不具任何申請效力！！
請病患妥為保管，若不慎遺失需自負個人資料外洩責任！
